# Increased Serum Sodium and Serum Osmolarity Are Independent Risk Factors for Developing Chronic Kidney Disease; 5 Year Cohort Study

**DOI:** 10.1371/journal.pone.0169137

**Published:** 2017-01-12

**Authors:** Masanari Kuwabara, Ichiro Hisatome, Carlos A. Roncal-Jimenez, Koichiro Niwa, Ana Andres-Hernando, Thomas Jensen, Petter Bjornstad, Tamara Milagres, Christina Cicerchi, Zhilin Song, Gabriela Garcia, Laura G. Sánchez-Lozada, Minoru Ohno, Miguel A. Lanaspa, Richard J. Johnson

**Affiliations:** 1 University of Colorado Denver, School of Medicine, Division of Renal Diseases and Hypertension, Aurora, Colorado, United States of America; 2 Toranomon Hospital, Department of Cardiology, Tokyo, Japan; 3 St. Luke’s International Hospital, Cardiovascular Center, Tokyo, Japan; 4 Tottori University Graduate School of Medical Sciences, Division of Regenerative Medicine and Therapeutics, Yonago, Japan; 5 Department of Pediatric Endocrinology, University of Colorado School of Medicine, Aurora, Colorado, United States of America; 6 Laboratory of Renal Physiopathology and Nephrology Dept, Instituto Nacional de Cardiología Ignacio Chávez, Mexico; Istituto Di Ricerche Farmacologiche Mario Negri, ITALY

## Abstract

**Background:**

Epidemics of chronic kidney disease (CKD) not due to diabetes mellitus (DM) or hypertension have been observed among individuals working in hot environments in several areas of the world. Experimental models have documented that recurrent heat stress and water restriction can lead to CKD, and the mechanism may be mediated by hyperosmolarity that activates pathways (vasopressin, aldose reductase-fructokinase) that induce renal injury. Here we tested the hypothesis that elevated serum sodium, which reflects serum osmolality, may be an independent risk factor for the development of CKD.

**Methods:**

This study was a large-scale, single-center, retrospective 5-year cohort study at Center for Preventive Medicine, St. Luke’s International Hospital, Tokyo, Japan, between 2004 and 2009. We analyzed 13,201 subjects who underwent annual medical examination of which 12,041 subjects (age 35 to 85) without DM and/or CKD were enrolled. This analysis evaluated age, sex, body mass index, abdominal circumference, hypertension, dyslipidemia, hyperuricemia, fasting glucose, BUN, serum sodium, potassium, chloride and calculated serum osmolarity.

**Results:**

Elevated serum sodium was an independent risk factor for development of CKD (OR: 1.03, 95% CI, 1.00–1.07) after adjusted regression analysis with an 18 percent increased risk for every 5 mmol/L change in serum sodium. Calculated serum osmolarity was also an independent risk factor for CKD (OR: 1.04; 95% CI, 1.03–1.05) as was BUN (OR: 1.08; 95% CI, 1.06–1.10) (independent of serum creatinine).

**Conclusions:**

Elevated serum sodium and calculated serum osmolarity are independent risk factors for developing CKD. This finding supports the role of limiting salt intake and preventing dehydration to reduce risk of CKD.

## Introduction

Epidemics of chronic kidney disease (CKD) [defined as an estimated glomerular filtration rate (eGFR) of < 60 ml/min/1.73 m^2^] not due to diabetes mellitus (DM) or hypertension have recently been observed among individuals working in hot environments in several areas of the world, including Central America, Mexico, India and Sri Lanka.[1–3] While the etiology is not known, a common risk factor is heat stress and recurrent dehydration, leading some authors to suggest that the disease should be called ‘heat stress nephropathy”.[3, 4] Experimental models have confirmed that recurrent heat stress and water restriction can lead to CKD, and the mechanism appears to be mediated by hyperosmolarity that activates pathways (vasopressin, aldose reductase-fructokinase) that can induce renal injury.[5–8]

If transient but recurrent hyperosmolarity is a risk factor for CKD, then evidence for such a mechanism might also be present in the general population. Indeed, there have been a number of studies that have suggested that a low fluid intake may be a risk factor for CKD [9–12] while others could not demonstrate such an association or showed the converse.[13, 14] However, the type of fluid intake may be very important, as some studies suggest that intake of water is protective but not when other drinks (such as soft drinks) are evaluated.[12] This is consistent with experimental models in which intake of water can protect against kidney injury whereas sugary beverages accelerate injury.[6, 15]

We therefore decided to test the hypothesis that an elevated serum osmolarity might be an independent predictor of CKD in a longitudinal study of Japanese adults. As an elevated serum osmolarity is strongly associated with an increase in serum sodium, we performed our primary analysis to determine if serum sodium is a predictor for CKD, as well as calculated serum osmolarity and its other components (BUN and glucose).

## Materials and Methods

### Study design and study subjects

This study was a large-scale, single-center, retrospective cohort study to clarify risk factors for developing CKD. We used the database at the Center for Preventive Medicine, St. Luke’s International Hospital, Tokyo, Japan. We analyzed the medical records of study subjects who underwent annual regular health check-up for general population at the center both in 2004 and 2009. When the study subjects had examinations more than once a year, we used only the first results in the same year to avoid double count. While all the population was able to access to the center, these medical examinations were out of insurance. Every subject and/or their companies paid for the examinations and each subject had identical physical and laboratory examinations. Serum creatinine was measured by enzymatic analysis and was calibrated to an isotope-dilution mass spectrometry (IDMS) standard. Serum Sodium was measured by ion-selective electrode measurements. The samples were measured using the BioMajesty^TM^ (NIHON KOHDEN Corporation, Tokyo, Japan) auto-analyzer. All blood samples were collected in the morning and performed in the same laboratory. Our population was ‘an apparently healthy population’ as they came to the center to have annual regular health check-up by themselves, and also provided a general history for comorbidities. Our study design allowed us to identify the development of CKD in apparently healthy people.

The study included subjects between 30 years and 85 years old at the 2004 examination. We excluded subjects with CKD in 2004 (baseline) because the study objective was to clarify risk factors for the development of new CKD. Furthermore, we excluded subjects with DM in 2004 because high blood glucose had large influences to the calculated osmolarity and DM is an established risk factor for CKD.

### Definition of CKD, DM, hypertension, dyslipidemia, hyperuricemia, and serum osmolarity

CKD was defined as the subjects whose eGFR of < 60 ml/min/1.73 m^2^ by calculated using the Japanese GFR equation; *eGFR (ml/min/1*.*73 m*^*2*^*) = 194 × Serum creatinine*^*-1*.*094*^ *× age*^*-0*.*287*^ *(×0*.*739 if women)*.[16] This is considered the classical definition for CKD in Japan. In this study, we defined CKD based on one time measurements of eGFR <60 ml/min/1.73m^2^ both in 2004 and in 2009. The serum creatinine was measured by enzymatic analysis and was calibrated to an IDMS standard. DM was defined as the subjects who had current history of DM and/or HbA1c [National Glycohemoglobin Standardization Program (NGSP)] of ≥6.5%. Hypertension was defined as the subjects who had current medication for hypertension and/or whose systolic blood pressure (BP) of ≥140 mmHg and/or diastolic BP of ≥90 mmHg. BP readings were obtained using an automatic brachial sphygmomanometer (OMRON healthcare Co., Ltd, Kyoto, Japan). Two readings were taken, after the participant had been seated and resting quietly for more than five minutes, with the feet on the ground and back supported. Mean systolic and diastolic BP for each participant was calculated from the recorded measurements. Dyslipidemia was defined as the subjects who had current medication for dyslipidemia and/or whose low-density lipoprotein cholesterol level of ≥140 mg/dl, high-density lipoprotein cholesterol level of <40 mg/dL, and/or triglyceride level of ≥150 mg/dL. Hyperuricemia is defined the subjects who had current medication for hyperuricemia and/or whose serum uric acid level >7.0 mg/dL. This definition was from Japanese guideline for the management of hyperuricemia and gout: second edition.[17] Serum osmolarity (mOsm/L) was calculated using a formula that takes into account serum sodium, BUN, and glucose; *(2×Sodium) + (BUN/2*.*8) + (Glucose/18)*.[18]

### Statistical analysis

We calculated the cumulative incidence rates of CKD over 5 years for each serum sodium level in 2004. The risk factors for the development of CKD were evaluated by logistic regression analyses. The regression analyses were adjusted for age, sex, body mass index (BMI), abdominal circumference, hypertension, dyslipidemia, hyperuricemia, fasting glucose, BUN, serum sodium, potassium and chloride. Furthermore, we also conducted logistic regression analyses with calculated serum osmolarity after adjusted for age, sex, BMI, abdominal circumference, hypertension, dyslipidemia, and hyperuricemia.

Calculated serum osmolarity differed between men and women (**Table 1**), and multivariable regression analyses were also stratified by sex. These logistic regression analyses modeled serum sodium and calculated osmolarity level in 2 ways—as a continuous variable and by quartiles. We calculated and compared the cumulative incidence rates of developing CKD in each serum sodium quartile and calculated osmolarity quartile by using logistic regression models. Statistical differences among serum sodium and calculated osmolarity quartiles were evaluated by using logistic regression analyses on these quartiles, with the lowest quartile as the reference group.

**Table 1:** Study subjects’ demographic data by sex in 2004 (baseline).

		Total	Men	Women	p
Number of subjects		12,041	5,598	6,443	
Age	years old	50.2±11.0	51.2±10.8	49.3±10.8	< 0.001
Height	Cm	163.3±8.6	170.0±6.1	157.5±5.6	< 0.001
Weight	Kg	60.0±11.8	68.7±9.7	52.5±7.6	< 0.001
Body mass index	kg/m^2^	22.4±3.1	23.7±2.8	21.2±2.9	< 0.001
Abdominal circumference	Cm	81.0±11.7	84.9±7.8	77.5±13.3	< 0.001
Systolic blood pressure	mmHg	118.3±17.7	123.3±16.8	114.0±17.3	< 0.001
Diastolic blood pressure	mmHg	73.7±11.3	77.4±10.8	70.6±10.9	< 0.001
Pulse rate	Bpm	73.4±10.7	71.3±10.2	75.2±10.8	< 0.001
Hypertension	%	17.8	24.1	12.3	< 0.001
Dyslipidemia	%	37.5	48.2	24.2	< 0.001
Hyperuricemia	%	13.6	28.2	0.9	< 0.001
Fasting glucose	mg/dL	98.0±9.2	101.7±9.2	94.8±7.9	< 0.001
Blood urea nitrogen	mg/dL	13.8±3.2	14.4±3.1	13.3±3.2	< 0.001
Serum sodium	mmol/L	141.6±1.8	141.8±1.7	141.3±1.8	< 0.001
Serum potassium	mmol/L	4.17±0.28	4.23±0.28	4.12±0.28	< 0.001
Serum chloride	mmol/L	105.9±1.8	105.8±1.9	106.0±1.8	< 0.001
Serum uric acid	mg/dL	5.3±1.4	6.2±1.2	4.5±0.9	< 0.001
Serum osmolality	mOsmol/kg	293.5±4.0	294.4±3.5	292.7±4.2	< 0.001
Estimated GFR	ml/min/1.73 m2	86.7±14.7	84.1±13.9	89.0±15.0	< 0.001
Proteinuria*	%	0.142%	0.197%	0.094%	< 0.001

GFR: glomerular filtration rate, mOsm/L: osmolarity per little, bpm: beats per minute, p: probability. Values are expressed as mean ± standard deviation. All items had significant difference between men and women (p < 0.001)

* We could not collect 87 urine samples for proteinuria measurement (3 men, 84 women). Proteinuria was measured by urinary test-strip. Values of ± correlate approximately to 15 mg/dL; + with 30 mg/dL; 2+ with 100 mg/dL; and 3+ with 250 mg/dL of proteinuria. We defined 2+ and 3+ as abnormal proteinuria in this study.

All statistical analyses were performed using the SPSS Statistics software (IBM SPSS Statistics version 22 for Windows; IBM, New York). The statistically significant level was set at α = 0.05. All of the statistical analyses were two-sided. Bivariate associations between demographic and clinical characteristics were compared between men and women using *t*-tests and χ^2^ analyses.

### Ethical considerations

All data were collected and compiled in a protected computer database. Individual data were anonymized and there was no personality information identified. St. Luke’s International Hospital Ethics Committee (9–1 Akashi-cho, Chuo-ku, Tokyo 104–8560, Japan) approved the protocol for this study. We had consents from all the subjects by comprehensive agreement method in the hospital.

## Results

### Characteristics of study subjects

**Fig 1:**
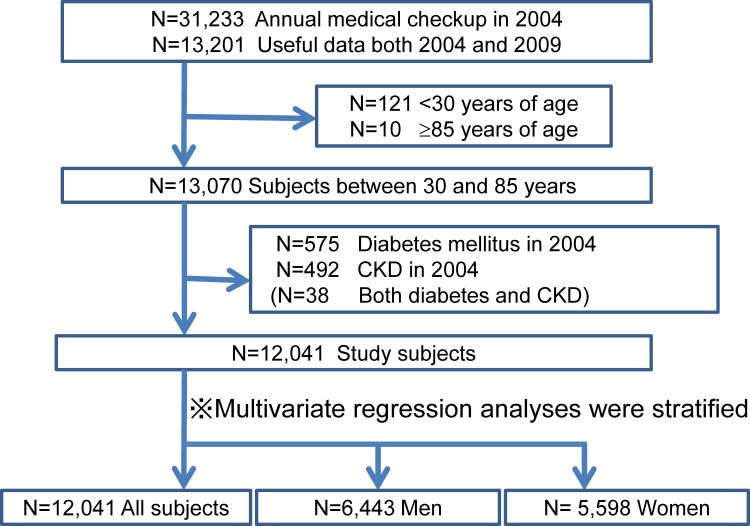
Flow diagram of study enrollment. Of 13,201 subjects who underwent annual medical examinations at the center in 2004 and again in 2009, we enrolled 12,041 subjects (6,443 men) between 30 years and 85 years old without CKD and DM in 2004.

We retrospectively analyzed the medical records of 13,201 subjects who underwent annual medical examinations at the center in 2004 and again in 2009. Of those, 13,070 subjects met the age requirement of being between 30 years and 85 years old at the initial (2004) examination. We excluded 492 subjects with preexistent CKD and/or 575 subjects with DM (38 both) that was present on presentation in 2004. Ultimately 12,041 subjects without CKD and DM in 2004 were enrolled in order to identify risk factors for developing CKD by using those follow-up data over a 5 year period (**Fig 1**). Baseline demographic data on the study subjects are shown in **Table 1**.

The prevalence of elevated serum sodium (defined as the highest quartile, or serum sodium >143 mmol/L) was 32.2% in men and 26.4% in women. The cumulative incidence (rate) of new CKD from 2004 to 2009 was 970 (17.3%) in men and 868 (13.5%) in women.

### Cumulative incidence rates of CKD in each serum sodium level

**Fig 2:**
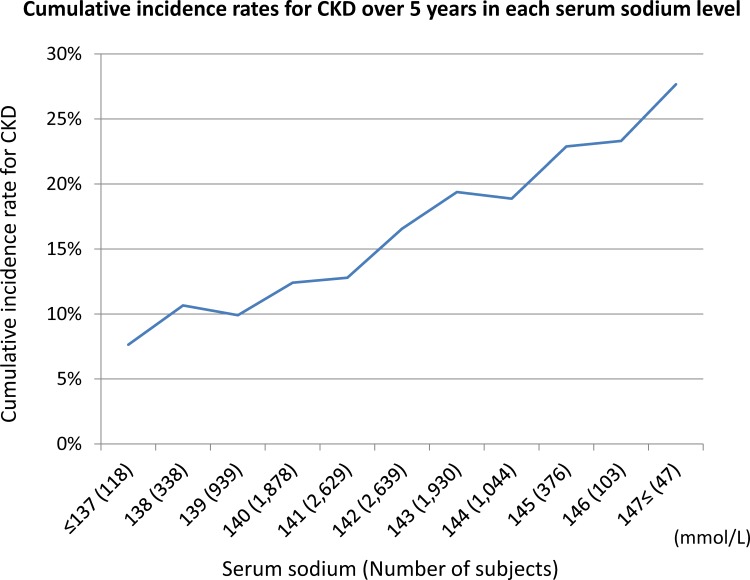
Cumulative incidence rates of CKD over 5 years in each serum sodium level in 2004. Higher serum sodium in 2004 reflected higher cumulative incidence rates of CKD over 5 years.

The cumulative incidence rates of CKD over 5 years relative to baseline serum sodium level in 2004 was shown in **Fig 2**. This graph showed a remarkable relationship between serum sodium in 2004 and cumulative incidence rates of CKD

### Risk factors for developing CKD

After multiple adjustments, the risk factors for developing CKD were as follows: aging [Odds Ratio (OR) per 1 year increased: 1.07; 95% confidence interval (CI), 1.06–1.07], higher BMI (OR: 1.12 per 1 kg/m^2^ increased; 95% CI, 1.08–1.16), smaller abdominal circumference (OR: 0.97 per 1cm increased; 95% CI, 0.96–0.98), hypertension (OR:1.23; 95% CI, 1.08–1.41), dyslipidemia (OR:1.14; 95% CI, 1.02–1.28), hyperuricemia (OR: 1.67; 95% CI, 1.44–1.95), lower fasting glucose (OR:0.99 per 1 mg/dL increased; 95% CI, 0.98–0.99), higher BUN (OR per 1 mg/dL increased: 1.08; 95% CI, 1.06–1.10), and higher serum sodium (OR: 1.03 per 1 mmol/L increased, 95% CI, 1.00–1.07) (**Table 2**). Elevated serum sodium was an independent risk factor for development of CKD with an 18 percent increased risk for every 5 mmol/L change in serum sodium. Baseline serum potassium and chloride were not risk factors for developing CKD after multiple adjustments.

**Table 2:** Risk factors for chronic kidney disease between 2004 and 2009.

		Crude			Adjusted*	
	Odds ratio	95% CI	P	Odds ratio	95% CI	p
Age (per 1 year older)	1.071	1.066–1.076	<0.001	1.065	1.059–1.071	<0.001
Gender (men vs women)	1.346	1.219–1.487	<0.001	0.959	0.846–1.087	0.521
Body mass index (per 1 kg/m^2^ increased)	1.061	1.045–1.077	<0.001	1.120	1.081–1.160	<0.001
Abdominal circumference (per 1 cm increased)	1.018	1.012–1.023	<0.001	0.967	0.955–0.979	<0.001
Hypertension (positive vs negative)	2.185	1.949–2.448	<0.001	1.233	1.081–1.407	0.002
Dyslipidemia (positive vs negative)	1.623	1.469–1.794	<0.001	1.144	1.023–1.279	0.018
Hyperuricemia (positive vs negative)	1.822	1.604–2.070	<0.001	1.673	1.436–1.950	<0.001
Fasting glucose (per 1 mg/dL increased)	1.016	1.011–1.022	<0.001	0.987	0.981–0.994	<0.001
Blood urea nitrogen (per 1 mg/dL increased)	1.154	1.137–1.172	<0.001	1.081	1.063–1.100	<0.001
Serum sodium (per 1 mmol/L increased)	1.162	1.129–1.195	<0.001	1.034	1.001–1.069	0.043
Serum potassium (per 1 mmol/L increased)	1.624	1.367–1.929	<0.001	1.143	0.954–1.389	0.143
Serum chloride (per 1 mmol/L increased)	1.061	1.032–1.091	<0.001	1.028	0.997–1.060	0.080

CI: confidence interval, p: probability.

*Data was adjusted for age, sex, body mass index, abdominal circumference, hypertension, dyslipidemia, hyperuricemia, fasting glucose, blood urea nitrogen, sodium, potassium, and chloride.

In calculated serum sodium quartiles, consisting of serum sodium level of ≤140 (n = 3,273), 141 (n = 2,629), 142 (n = 2,639), and ≥143 (n = 3,500), the highest quartile had a 1.17-fold risk for CKD (95% CI, 1.038–1.390) compared with the lowest quartile after adjustments for age, sex, BMI, abdominal circumference, hypertension, dyslipidemia, hyperuricemia, fasting glucose, and BUN (**Table 3**).

**Table 3:** Relative odds ratio of chronic kidney disease stratified among serum sodium quartile between 2004 and 2009.

Serum sodium quartile (mmol/L)			Crude			Adjusted*	
	N	Odds ratio	95% CI	P	Odds ratio	95% CI	P
1 (≤140)	3,273	Reference			Reference		
2 (141)	2,629	1.146	0.979–1.342	0.089	0.931	0.789–1.099	0.396
3 (142)	2,639	1.552	1.337–1.802	<0.001	1.065	0.909–1.247	0.437
4 (143≤)	3,500	1.935	1.688–2.217	<0.001	1.168	1.009–1.352	0.038

C.I.: confidence interval, p: probability.

*Data adjusted for age, sex, body mass index, abdominal circumference, hypertension, dyslipidemia, hyperuricemia, fasting glucose, and BUN.

**Fig 3:**
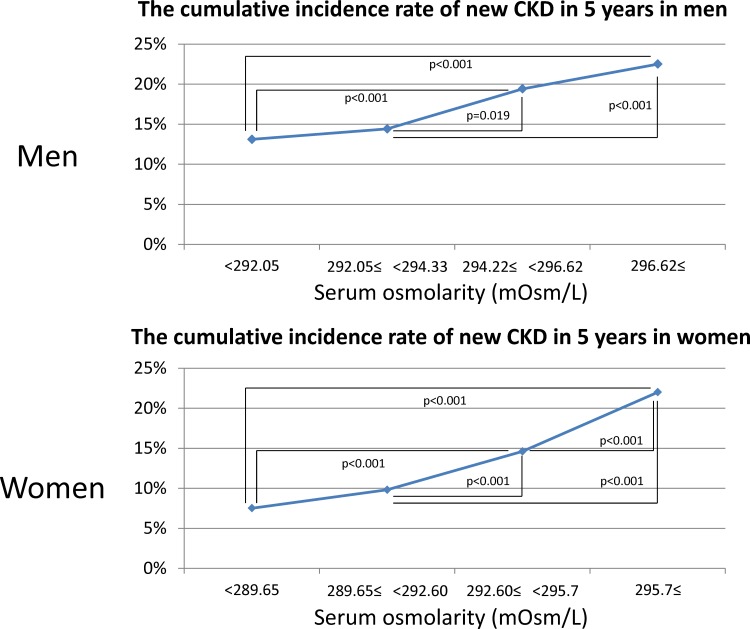
The cumulative incidence rate of new chronic kidney disease by quartile of serum osmolarity. After using logistic regression model, there were significant differences of the cumulative incidence rate of new chronic kidney disease between quartile 1 and 3, 4, quartile 2 and 3, 4 in men, between quartile 1 and 3, 4, quartile 2 and 3, 4, and quartile 3 and 4 in women (p<0.05).

Calculated serum osmolarity was also a significantly independent risk factor for developing CKD after multiple adjustments (OR: 1.04 per 1 mOsm/L increased; 95% CI, 1.025–1.054) (**Table 4**). For every change in 5 mOsm/L, there was an increased risk of 24 percent for development of CKD. After conducting similar analyses by sex, higher calculated serum osmolarity remained a significantly independent risk factor for developing CKD both in men (OR: 1.05 per 1 mOsm/L increased; 95% CI, 1.03–1.07) and women (OR: 1.03 per 1 mOsm/L increased; 95% CI, 1.01–1.05). Higher quartiles of serum osmolarity also carried higher incidence rates of new CKD for both men and women (**Fig 3**). In men, study subjects in the highest serum osmolarity quartile had a 1.72-fold higher cumulative incidence rate of new CKD compared to those in the lowest quartile (22.5% versus 13.1%, p<0.001). In women, subjects in the highest serum osmolarity quartile had a 2.93-fold higher cumulative incidence rate of new CKD compared to those in the lowest quartile (22.0% versus 7.5%, p<0.001). After using a logistic regression model, there were significant differences between quartile 1 and 3, 4, quartile 2 and 3, 4 in men, between quartile 1 and 3, 4, quartile 2 and 3, 4, and quartile 3 and 4 in women (**Fig 3**). After adjustments for age, BMI, abdominal circumference, hypertension, dyslipidemia, and hyperuricemia, these ORs were 1.52 (95% CI, 1.23–1.88) in men and 1.34 (95% CI, 1.04–1.72) in women (**Table 5**).

**Table 4:** Calculated serum osmolarity as a risk factor for chronic kidney disease between 2004 and 2009.

			Crude			Adjusted*	
		Odds ratio	95% CI	P	Odds ratio	95% CI	P
Total	Serum osmolarity	1.107	1.092–1.121	<0.001	1.039	1.025–1.054	<0.001
Men	Serum osmolarity	1.079	1.058–1.100	<0.001	1.052	1.031–1.074	<0.001
Women	Serum osmolarity	1.123	1.103–1.143	<0.001	1.032	1.010–1.053	0.003

CI: confidence interval, p: probability.

*Data adjusted for age, body mass index, abdominal circumference, hypertension, dyslipidemia, and hyperuricemia.

**Table 5:** Relative odds ratio of chronic kidney disease stratified among serum osmolarity quartile by sex between 2004 and 2009.

	Serum osmolarity quartile (mOsm/L)			Crude			Adjusted*	
		N	Odds ratio	95% CI	P	Odds ratio	95% CI	P
Men	1 (<292.05)	1,401	Reference			Reference		
	2 (292.05≤<294.33)	1,398	1.118	0.901–1.386	0.312	1.037	0.827–1.300	0.752
	3 (294.33≤<296.62)	1,402	1.602	1.306–1.965	<0.001	1.456	1.175–1.805	0.001
	4 (296.62≤)	1,397	1.930	1.580–2.357	<0.001	1.523	1.233–1.881	<0.001
Women	1 (<289.65)	1,615	Reference			Reference		
	2 (289.65≤<292.60)	1,610	1.344	1.049–1.721	0.019	1.008	0.780–1.302	0.952
	3 (292.60≤<295.70)	1,610	2.110	1.675–2.659	<0.001	1.037	0.804–1.337	0.778
	4 (295.70≤)	1,608	3.486	2.798–4.342	<0.001	1.336	1.037–1.721	0.025

mOsm/L: osmolarity per little, N: number of subjects, CI: confidence interval, p: probability.

*Data adjusted for age, body mass index, abdominal circumference, hypertension, dyslipidemia, and hyperuricemia.

### Relationship between calculated serum osmolarity and eGFR

**Fig 4:**
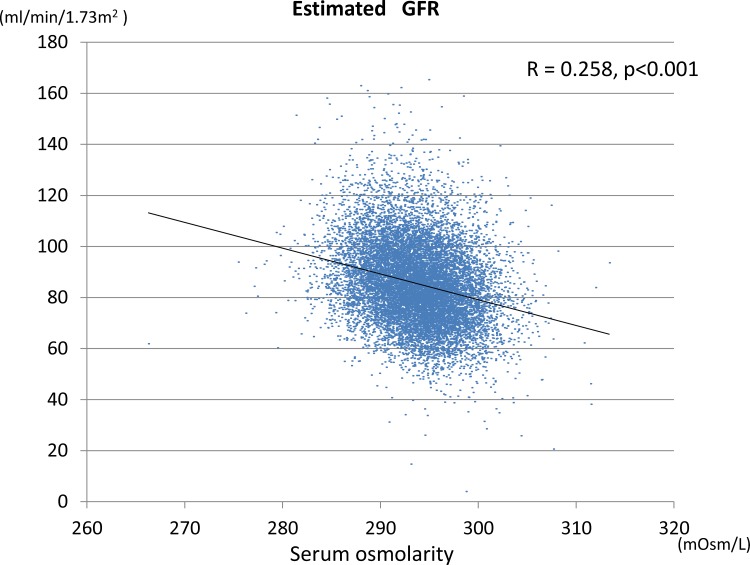
The correlation between serum osmolarity and kidney function. Pearson’s correlation analysis demonstrated an inverse correlation (p<0.001) between serum osmolarity and kidney function (eGFR) with an R value of -0.258.

Higher calculated serum osmolarity also independently predicted CKD as defined using the modified eGFR equation (**Fig 4**).

## Discussion

We tested the hypothesis that an elevated serum sodium might be a risk factor for CKD, as serum sodium generally reflects serum osmolarity. The hypothesis is based on the fact that an elevated serum osmolarity can activate various metabolic processes, including vasopressin release and activation of the aldose reductase-fructokinase pathway that can be associated with renal injury.[15, 19] We also evaluated other components of serum osmolarity, including fasting glucose and BUN levels. Our primary finding was that both elevated serum sodium and elevated calculated serum osmolarity were strongly associated with the development of CKD, with a 5 mmol/L change in serum sodium carrying 18 percent increased risk for CKD and with a 5 mOsm/L change in serum osmolarity carrying 24 percent increased risk. The increased risk was independent of multiple other known risk factors, including age, sex, BMI, abdominal circumference, hypertension, dyslipidemia, and hyperuricemia. Thus, our study presents the first evidence that elevated serum sodium and osmolarity may be independent risk factors for CKD.

We also found that an elevated BUN was also associated with increased risk for CKD which remained an independent risk factor after correcting for baseline eGFR. Since dehydration is commonly associated with an increase in the BUN/creatinine ratio, it is possible that the elevated BUN reflects a dehydrated state. Alternatively, it might mark subtle differences in GFR that were not detected using the eGFR calculated methods.

An unusual finding was that, while both fasting glucose and abdominal circumference predicted the development of CKD by crude analysis, that following multiple adjustments with other risk factors that the relationship switched to be inverse, in which both lower fasting glucose or lower abdominal circumference predicted the development of CKD (Table 2). It is possible that this reflects the consequence of multiple adjustments for factors that are causally linked with each other such as BMI, dyslipidemia and serum uric acid [20]. It is known that when factors are causally linked, that multivariable analysis may result in the factors not being independent or even showing opposing relationships. For example, the risk from an increased abdominal circumference may be nulled by controlling for BMI if the two factors are causally linked, and in fact if the effect of BMI. Likewise, since diabetes was excluded at baseline, the finding that an elevated fasting glucose was now associated with protection may reflect a risk with low fasting glucose that might reflect poor nutrition. Importantly, an elevated serum sodium remained strongly predictive of CKD in both crude analysis and following multiple risk factor adjustments.

This study supports the hypothesis that hyperosmolarity may have a role in the pathogenesis of CKD.[7] Specifically, we found both an increase in serum sodium and an elevated calculated serum osmolarity to predict the development of CKD. Hyperosmolarity stimulates vasopressin synthesis and release, the latter which can be assessed in the circulation by the presence of copeptin, a precursor for vasopressin.[21] Serum copeptin levels are associated with the presence of microalbuminuria and also predict progression of kidney disease in renal transplant recipients.[22–24] Experimentally vasopressin has been shown to accelerate CKD by causing glomerular hyperfiltration and albuminuria.[15, 25, 26] An increase in serum osmolarity also activates the polyol (aldose reductase) pathway that can ultimately lead to intrarenal fructose generation and tubular injury.[5] Heat stress and water restriction may lead to transient injury to the kidney (acute kidney injury) that then leads to CKD.[27–29] These studies, along with epidemiological studies linking low water intake with risk for CKD,[10, 12] provide a mechanistic pathway between dehydration and CKD, and has led to a clinical trial to determine if increasing water intake can slow the progression of CKD.[30]

Strengths of the study included its longitudinal nature, and the large subject population. The restriction of subjects to age 30 to 85 was because subjects over 85 years old had a high possibility of death and subjects under 30 years old had low possibility of new onset of CKD. We also excluded subjects with DM since it is a strong risk factor for CKD.[31, 32]

The study has several limitations. First, serum osmolarity was calculated rather than directly measured.[18, 33] We could not measure serum osmolality directly because this study was retrospective and the original samples were no longer available. However, we did have direct measurements of serum sodium, which also was an independent risk factor for CKD. Second, this study conducted only one time measurements of serum sodium at 2004 and 2009. The study conducted a two-point cohort study between in 2004 and in 2009 by matching the study subjects identification numbers, and we could not access data between 2005 to 2008. However, all the blood testing was conducted in the morning, and the consistency in timing makes it more translatable across the sample population. In addition, the relationship between baseline serum uric acid and cumulative incidence of CKD was linear, suggesting a strong relationship between the baseline value and outcome. Our study was also a retrospective, single-center study and therefore may have a selection bias. The study group was an apparently healthy population and they came to the center for their health check-up by themselves, and there was little referral bias. In addition, while we analyzed almost all subjects who had annual examination both in 2004 and 2009, there were a large number of subjects who were examined in 2004 who did not return in 2009 for follow-up, and we do not have details of this group. Nevertheless, a comparison of subjects who returned in 2009 (n = 12,041) compared to those that did not return (n = 16,705) suggest similarities in BMI (22.4 vs 22.5 kg/m^2^), blood pressure (118/74 vs 118/73 mmHg), fasting glucose (98.0 vs 99.8 mg/dL), BUN (13.8 vs 13.7 mg/dL), and serum sodium (141.8 vs 141.5 mmol/L). In addition, CKD generally requires confirmatory documentation for reduced eGFR of <60 ml/min/1.73m^2^ at 3 months, but our study only collected data once each year because they were apparently general healthy people. A strength of the data, however, is that all subjects that were followed had identical laboratory tests performed with the same laboratory.

In addition, while we knew who was receiving medications, the specific medications being taken were not available. However, the number of study subjects who were on medications were low, and for hypertension was 1,006 (8.6%), for dyslipidemia was 508 (4.2%) and for hyperuricemia and/or gout was 264 (2.2%) in 2004. Since serum sodium can be primary affected by diuretics, the primary concern would be the group with hypertension. Therefore, we repeated an analysis in which we excluded subjects with hypertension who were receiving medications (n = 1,006, 634 men). The results were almost same compared with Table 2, and serum sodium remained an independent risk for CKD with an OR of 1.045 (95% CI, 1.009–1.083). Finally, this study shows elevated serum sodium and calculated serum osmolarity independently predict CKD, but this does not mean this is a causal relationship. Controlled trials to determine if reducing or preventing elevated serum osmolarity slows or prevents CKD should be performed to test this hypothesis, and one randomized control trial is currently underway.[30]

## Conclusions

This study identifies higher sodium and calculated serum osmolarity as risk factors for CKD. This observation may be relevant not only to the role of inadequate water intake, but also to other dietary measures that may raise serum osmolarity, such as high salt intake [34] and fructose-containing soft drinks.[6] Further studies are indicated, but our work suggests the potential for simple lifestyle measures (increasing water intake and decreasing salt intake) as a means of protecting against the deterioration of kidney function.
