# Correction: Increased Serum Sodium and Serum Osmolarity Are Independent Risk Factors for Developing Chronic Kidney Disease; 5 Year Cohort Study

**DOI:** 10.1371/journal.pone.0197941

**Published:** 2018-05-17

**Authors:** Masanari Kuwabara, Ichiro Hisatome, Carlos A. Roncal-Jimenez, Koichiro Niwa, Ana Andres-Hernando, Thomas Jensen, Petter Bjornstad, Tamara Milagres, Christina Cicerchi, Zhilin Song, Gabriela Garcia, Laura G. Sánchez-Lozada, Minoru Ohno, Miguel A. Lanaspa, Richard J. Johnson

There is an error in Fig 1 and the Fig 1 caption. The N values for Men and Women are swapped. The correct values are N = 5,598 Men and N = 6,443 Women. Please see the correct Fig 1 and caption here.

**Fig 1:**
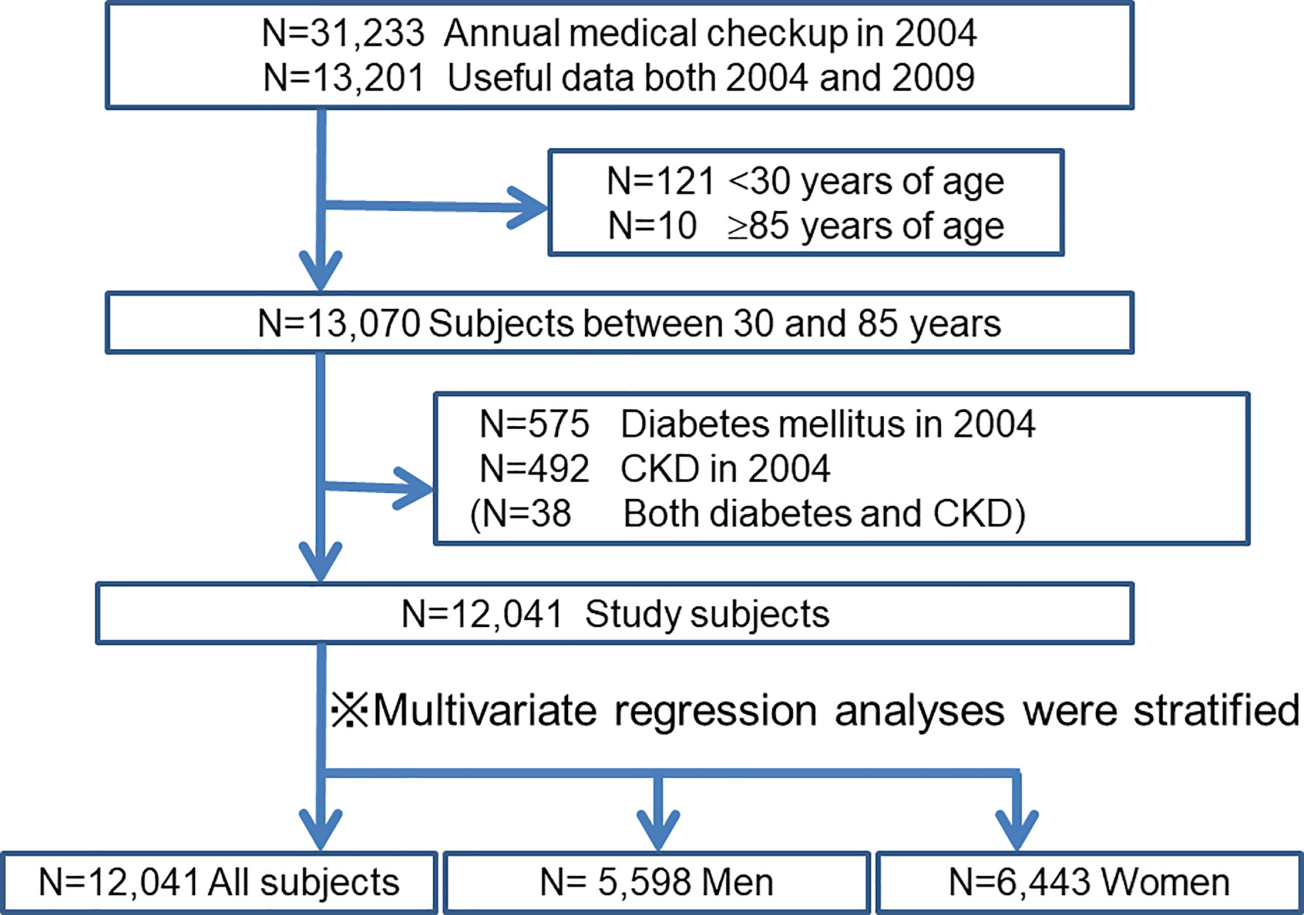
Flow diagram of study enrollment. Of 13,201 subjects who underwent annual medical examinations at the center in 2004 and again in 2009, we enrolled 12,041 subjects (5,598 men) between 30 years and 85 years old without CKD and DM in 2004.
